# BRAX, Brazilian labeled chest x-ray dataset

**DOI:** 10.1038/s41597-022-01608-8

**Published:** 2022-08-10

**Authors:** Eduardo P. Reis, Joselisa P. Q. de Paiva, Maria C. B. da Silva, Guilherme A. S. Ribeiro, Victor F. Paiva, Lucas Bulgarelli, Henrique M. H. Lee, Paulo V. Santos, Vanessa M. Brito, Lucas T. W. Amaral, Gabriel L. Beraldo, Jorge N. Haidar Filho, Gustavo B. S. Teles, Gilberto Szarf, Tom Pollard, Alistair E. W. Johnson, Leo A. Celi, Edson Amaro

**Affiliations:** 1 Hospital Israelita Albert Einstein – Big Data Analytics, São Paulo, Brazil; 2 Hospital Israelita Albert Einstein – Imaging Department, São Paulo, Brazil; 3 Massachusetts Institute of Technology – Laboratory for Computational Physiology, Cambridge, USA; 4 The Hospital for Sick Children – Peter Gilgan Centre for Research and Learning, Toronto, Canada; 5 Beth Israel Deaconess Medical Center – Department of Medicine, Boston, USA; 6 Harvard T.H. Chan School of Public Health – Department of Biostatistics, Boston, USA

**Keywords:** Diagnostic markers, Respiratory tract diseases, Radiography

## Abstract

Chest radiographs allow for the meticulous examination of a patient’s chest but demands specialized training for proper interpretation. Automated analysis of medical imaging has become increasingly accessible with the advent of machine learning (ML) algorithms. Large labeled datasets are key elements for training and validation of these ML solutions. In this paper we describe the Brazilian labeled chest x-ray dataset, BRAX: an automatically labeled dataset designed to assist researchers in the validation of ML models. The dataset contains 24,959 chest radiography studies from patients presenting to a large general Brazilian hospital. A total of 40,967 images are available in the BRAX dataset. All images have been verified by trained radiologists and de-identified to protect patient privacy. Fourteen labels were derived from free-text radiology reports written in Brazilian Portuguese using Natural Language Processing.

## Abstract (summary)

**Table:** 

Measurement(s)	Chest Radiography
Technology Type(s)	natural language processing
Factor Type(s)	radiological findings/labels
Sample Characteristic - Organism	Homo sapiens
Sample Characteristic - Environment	chest organ
Sample Characteristic - Location	Brazil

## Background & Summary

Chest radiographs are a major part of the imaging studies in hospitals worldwide, playing a fundamental role in the screening, diagnosis, and treatment of many pathologies^1^. Due to intensive work routines and the need for fast diagnoses, chest radiographs are often evaluated by the requesting physicians, who despite having received training in interpreting chest radiographs are not experts in their interpretation in the same manner as thoracic radiologists^2,3^. Moreover, the demand for the specialized evaluation of x-rays usually exceeds the available number of radiologists^4^. The use of Machine Learning (ML) algorithms to support clinical decisions has become increasingly popular in various radiology contexts^5,6^: workflow optimization^7^, detecting relevant imaging alterations to support disease diagnosis^8^, and also automated generation of radiology reports^9–11^. These solutions can be especially useful in underdeveloped regions and communities where there is a shortage of radiologists^12^. However, in order to develop ML solutions for radiology, high-quality annotation and a larger number of datasets are required to train and validate algorithms^13,14^. Geographic diversity – to account for demographic and phenotypic variation – is also particularly important to the generalizability of AI models^15^.

Various initiatives have been developed in recent years^12,16–19^, mainly including data from high-income countries and with reports written in English^15^. This is extremely relevant since Natural Language Processing (NLP) algorithms are heavily dependent on the language – i.e. the majority of NLP algorithms used for extraction of labels only work for English-based datasets (e.g., ChestX-ray8^17^, CheXpert^12^ and MIMIC-CXR^19^). Therefore, NLP solutions for other languages are required^16^.

Here we present BRAX, a dataset of labeled chest radiographs from a large general hospital in the region of São Paulo, Brazil. The BRAX dataset contains 40,967 images corresponding to 24,959 radiographic studies from 19,351 patients. The NLP solution used to extract the labels is largely based on the CheXpert labeler, which was adapted to detect negation and uncertainty in Portuguese, a language spoken by over 270 million people worldwide^20^. We hope this dataset can contribute to reducing the number of under-represented populations in the available pool of chest radiograph datasets used for the development of models for clinical decision support.

## Methods

**Fig. 1:**
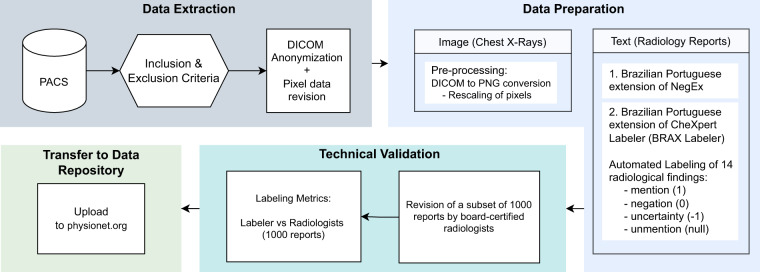
BRAX dataset creation flowchart. Data Extraction: Only chest radiographs accompanied by a radiology report were included. Images were anonymized and checked for burned-in sensitive data; Data Preparation: DICOM images were converted to PNG format and rescaled. 14 radiological findings were extracted from free-text reports written in Brazilian Portuguese, after adaptation of NegEX and CheXpert Label Extraction Algorithm. Technical Validation: The labeling was validated by board-certified radiologists. Transfer to Data Repository: BRAX dataset is available on Physionet^21,22^ at https://physionet.org/content/brax/1.1.0/.

Figure 1 provides an overview of the dataset generation process^21,22^.

### Data collection

#### Ethical statement

The project was approved by the Institutional Review Board of Hospital Israelita Albert Einstein (#35503420.8.0000.0071). Requirement for individual patient consent was waived. The study database was anonymized, with all identifiable patient information removed, including the dates of acquisition of the radiographs.

#### Data source

**Fig. 2:**
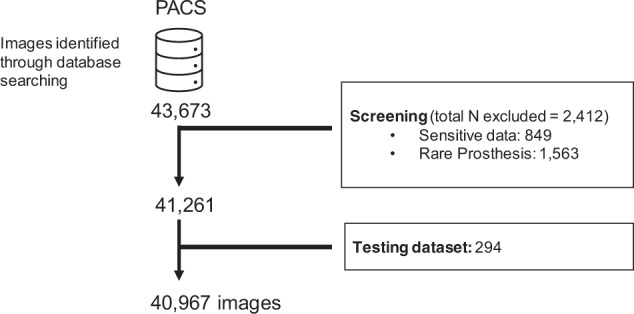
Flowchart detailing the BRAX dataset creation process. First, images were retrieved from the institutional PACS database. Next, exclusion criteria were applied, and then a subset was separated as a hidden test dataset.

All data was obtained from Hospital Israelita Albert Einstein (HIAE). Images were extracted from PACS (*Picture Archiving and Communication System*). All chest radiography studies with available reports in the institutional PACS were considered for inclusion. We selected 24,959 high-quality digital chest radiographic studies acquired prior to the COVID-19 pandemic. Radiographs with burned-in sensitive data (i.e., patient name, patient identity, and image display specifications) were excluded, as well as images with rare prosthesis that could facilitate patient identification. Figure 2 shows the BRAX dataset flowchart. A subset of 294 images was excluded from BRAX so that it could be used as a hidden test set for further evaluation of machine learning models. Those interested may run their models on this (not publicly available) subset, upon request to the corresponding author.

#### Anonymization procedure

DICOM header anonymization was accomplished using an algorithm developed in-house based on a previously described procedure^23,24^ and following the rules of the MIRC ClinicalTrialProcessor (CTP) DICOM Anonymizer^25^. The application removed DICOM metadata that could be used to identify patients, without compromising the relevant clinical information. We also added an extra conservative step by removing any free-text fields contained in the header. The fields *StudyDate*, *SeriesDate*, *AcquisitionDate* and *ContentDate* have been properly anonymized by a hashing procedure (i.e. fictitious dates), retaining only the original time intervals between study acquisitions, so that chronological information is not lost. Images were reviewed by a board-certified radiologist (E.P.R.) with over 2 years of experience to identify burned-in sensitive data according to the exclusion criteria mentioned above. The images were also double-checked by 5 other radiologists with up to 2 years of experience (M.C.B.S, H.M.H.L, V.M.B, L.T.W.A. and G.L.B.) in a way that each chest radiograph was reviewed by two radiologists in order to increase confidence in the application of exclusion criteria.

### Data preparation

#### Image preparation

All DICOM images were kept with the original uncompressed information and no transformation was applied in the space or contrast domains. In order to facilitate access to researchers, we used the open source SimpleITK^26^ python script^27^ to convert the DICOM images to PNG. The output image width was set to 1024 pixels, and grayscale images with high dynamic range were rescaled to [0,255] through intensity windowing (window width and window level were extracted from the DICOM metadata) before conversion to the new format. During rescaling, the intensity of the pixel values (obtained on the DICOM tag “PhotometricInterpretation”) is checked to determine whether they need to be inverted, so that air in the image appears white (highest pixel value), while the outside of the patient’s body appears black (lowest pixel value).

#### Radiology reports preparation

**Fig. 3:**
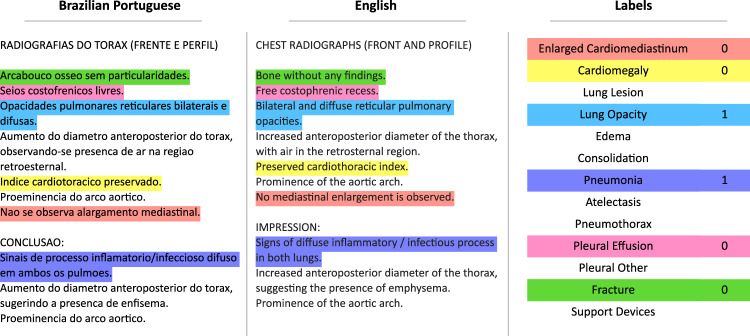
Automated labeling of the radiology reports. Example of the original radiology report in Brazilian Portuguese, its translation to English, and the final output of the automated labeling procedure.

All CXR images and reports were reviewed by at least one board-certified member of the radiology staff specialized in cardiothoracic imaging. To reduce inter-observer variability, reports - written in Brazilian Portuguese - are given in a standardized manner, according to the clinical indication. Radiology reports were originally stored in free-text form. Titled sections (i.e., detailed description of all *findings* and *impressions*) were based on templates. Example of a typical report is shown in Fig. 3A.

#### Automated labeling of the radiology reports

We implemented an automated extraction of labels from free-text radiology reports based on natural language processing. This process was based on two freely available tools: NegEx^28^ and CheXpert Label Extraction Algorithm^12^.

Brazilian Portuguese extension of NegEx for Detection of Negation and Uncertainty

1. We translated the NegEx trigger terms (i.e. a list of words that precede negation and uncertainty) from English to Brazilian Portuguese using the Google Sheet built-in function for Google Translate^29^ in order to speed up the process of human verification (Fig. 2B).
2. Three Brazilian radiologists reviewed the translated triggers and also included new ones to the NegEx lexicon, based on expressions related to negation and uncertainty specific to the radiology domain.

BRAX labeler: an expansion of the CheXpert Labeler for Brazilian Portuguese

1. The BRAX labeler was built upon CheXpert Labeler Algorithm^12^ to derive labeling from both the findings and impression sections of radiological reports written in Brazilian Portuguese (Fig. 3). Fourteen labels – Atelectasis, Cardiomegaly, Consolidation, Edema, Pleural effusion, Pneumonia, Pneumothorax, Enlarged cardiomegaly, Lung lesion, Lung opacity, Pleural other, Fracture, Support Devices, No Finding (Table 1) – representing the most common chest radiographic observations (Fig. 4), and used in previous studies^12,19^, were adapted to Brazilian Portuguese^30^. We have chosen to use the same labels from CheXpert^12^ because they have also been used in other large chest x-ray datasets, such as MIMIC-CXR^19^ and ChestX-Ray8^17^.

**Table 1:** Frequency of the radiological findings.

Pathology	Positive (%)	Uncertain (%)	Negative (%)
No findings	29009 (71.0)	0	11958 (29.0)
Enlarged Cardiom.	71 (0.17)	2 (0.00)	26212 (63.98)
Cardiomegaly	3984 (9.72)	0	28000 (68.35)
Lung Lesion	1290 (3.15)	19 (0.05)	46 (0.11)
Lung Opacity	4065 (9.92)	17 (0.04)	52 (0.13)
Edema	50 (0.12)	0 (0.0)	0 (0.0)
Consolidation	3157 (7.71)	0 (0.0)	19 (0.05)
Pneumonia	774 (1.89)	0 (0.0)	46 (0.11)
Atelectasis	3518 (8.59)	0 (0.0)	41 (0.10)
Pneumothorax	214 (0.52)	0 (0.0)	189 (0.46)
Pleural Effusion	1822 (4.45)	0 (0.0)	31422 (76.70)
Pleural Other	117 (0.29)	0 (0.0)	1 (0.00)
Fracture	624 (1.52)	0 (0.0)	16405 (40.04)
Support Devices	8791 (21.46)	0 (0.0)	21 (0.05)

The BRAX dataset consists of 14 labeled observations. We report the number of images which contain these observations.

**Fig. 4:**
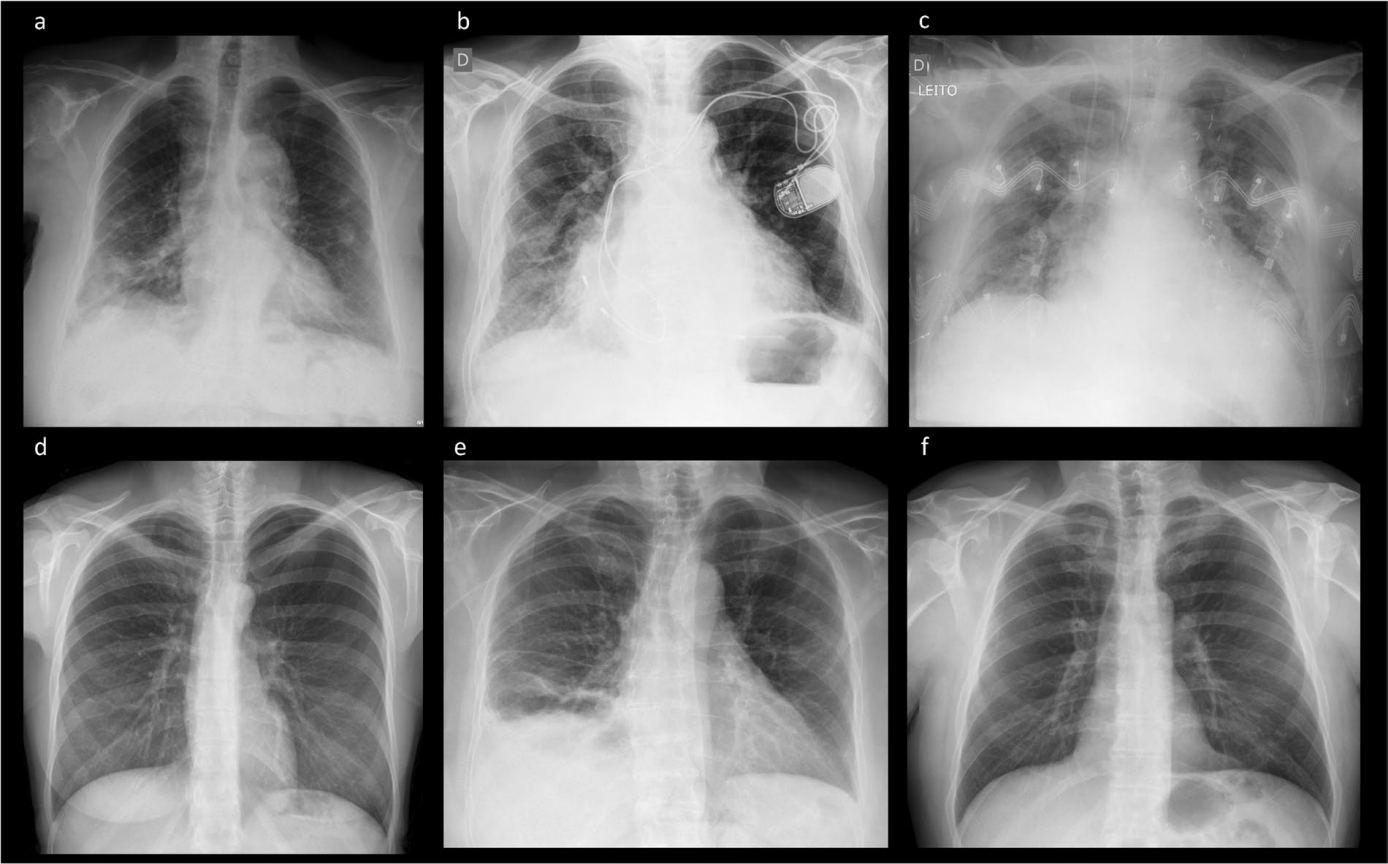
Example images included in the BRAX dataset. (**a**) Lung lesion, consolidation; (**b**) Cardiomegaly, device; (**c**) patient in intensive care bed, edema, cardiomegaly, device; (**d**) Pneumothorax; (**e**) pneumothorax, pleural effusion, consolidation, atelectasis; (**f**) No Findings.
2. Brazilian Portuguese radiological terms^30^ for each label were created based on CheXpert^12^ through an iterative process that involved a cardiothoracic radiologist (MCBS) and other general radiologists (EPR,HL) and then validated by senior cardiothoracic radiologists (GT, GS), according to the frequency and relevance of findings.
3. For each radiological finding reported, NegEx determines whether that label was in context of negation or uncertainty. This information is then coded (i.e. positive mention = 1, uncertain = −1, negation = 0, umention = null) to a CSV file (*master_spreadsheet.csv*), with one row per study and one column per finding.

## Data Records

**Fig. 5:**
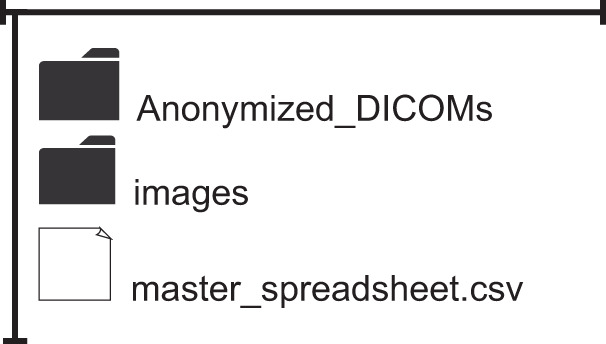
Folder structure of the BRAX dataset. The main repository contains two folders comprising the anonymized DICOM and PNG images respectively, in addition to the master spreadsheet, which contains the labels and the associated metadata for each image (DICOM/PNG).

BRAX dataset provides 40,967 images, 24,959 imaging studies for 19,351 patients presenting to the Hospital Israelita Albert Einstein. An overview of the released dataset folder structure is provided in Fig. 5. All data are available on PhysioNet^21,22^. Access is controlled and requires the user to register, complete a credentialing process, and sign a data use agreement (see usage notes). The BRAX project page on PhysioNet describes the dataset and informs users how they may apply for access.

### File organization

**Fig. 6:**
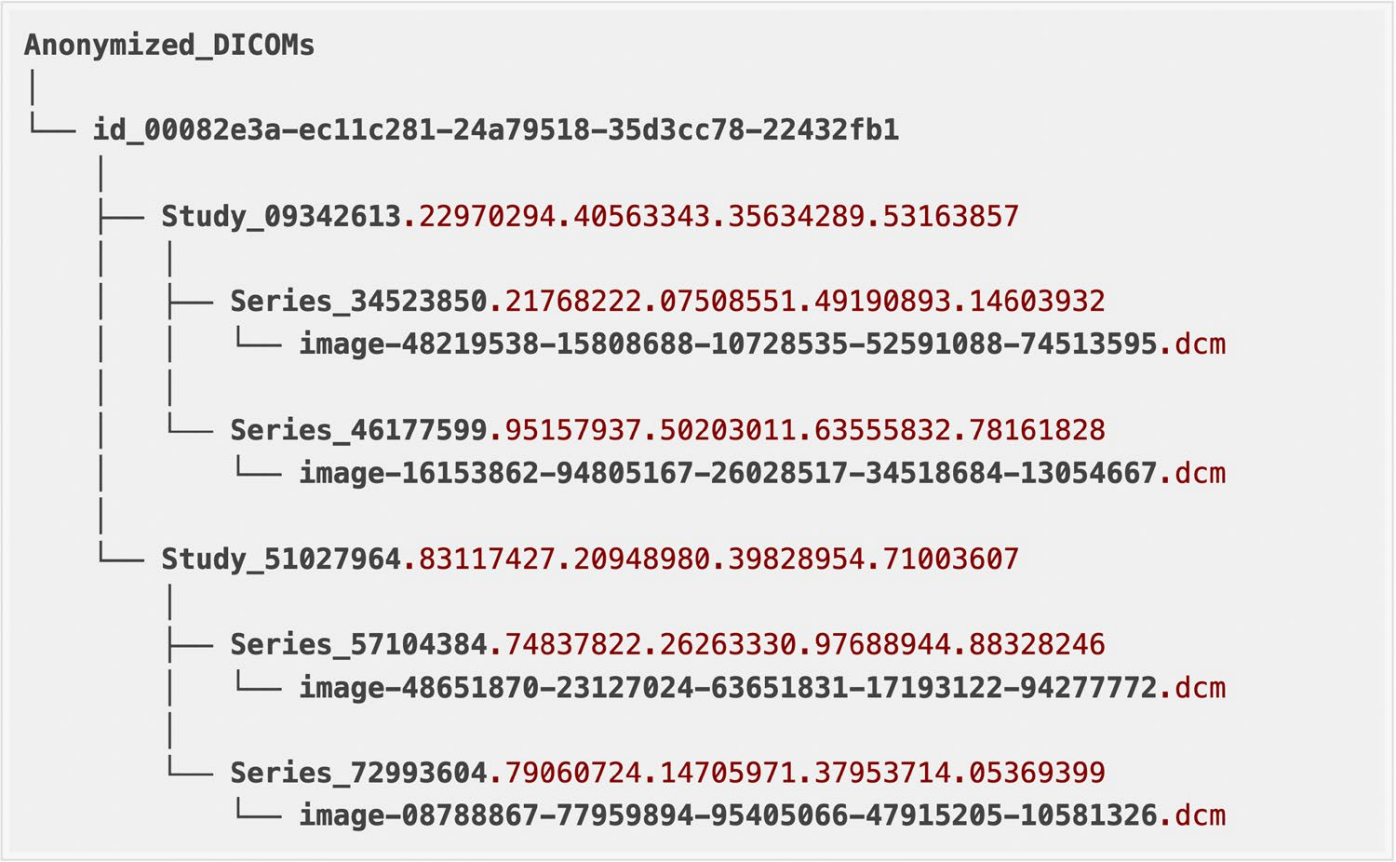
Example of the Anonymized_DICOMs folder structure for a single patient. Inside the main anonymized folder, subfolders are organized in the following hierarchy: patients (DICOM tag: *PatientID)*, studies (DICOM tag: *StudyInstanceUID)*, series (DICOM tag: *SeriesInstanceUID)*, and images (DICOM tag: *SOPInstanceUID*).

Image files are provided in individual folders. *PatientID* refers to the unique identifier for a single patient. The same patient can have multiple studies. A collection of images associated with a single report is referred to as a study and is identified by the *AccessionNumber*. Radiograph images in different view positions (usually frontal or lateral views) can be found in different or the same series depending on modality and how the DICOMs were generated during acquisition. An example of the Anonymized_DICOMs folder structure for a single patient’s images is provided in Fig. 6. The folder name starts with “id” followed by the number for the *PatientID* DICOM Tag. This example patient has two radiographic studies. The study folder name starts with *Study* followed by the number for the *StudyInstanceUID* DICOM Tag. Each study has one or more series folders, starting with *Series* followed by the number for the *SeriesInstanceUID* DICOM Tag. Finally, inside each series folder you may find one or more x-ray DICOM files, with the image file name starting with “image” followed by the number for the *SOPInstanceUID* DICOM Tag. All identifiers were randomly generated, and their order is not associated with the chronological order of the actual studies.

BRAX contains:

- “Anonymized_DICOMs” folder - all DICOM images organized in sub-folders according to patient identifier, study, series and image (see the section Folder Structure)
- “images” folder - the same structure as the Anonymized_DICOMs folder but containing PNG files instead of DICOM
- “master_spreadsheet.csv” - the main dataset table containing the identifiers for each image and associated metadata. The table provides one row per study and one separate column for each label. Columns are detailed below.

#### Description of columns in the master_spreadsheet.csv

*DicomPath:* Path to the DICOM images. As part of the de-identification procedure, the DICOM’s were assigned randomly generated ID numbers.

*PngPath:* Path to the PNG images.

*PatientID:* Patient’s identifier. As part of the de-identification procedure, the Patient IDs were created with randomly generated numbers.

*PatientSex:* Patient’s sex. Enumerated Values: “M” for male; “F” for female; “O” other.

*PatientAge:* Age of the patient is provided in 5-year groups. Patients aged 85 or over are classified as “85 or more”.

*AccessionNumber:* A DICOM identifier of the Study. As part of the de-identification procedure, the AccessionNumber was randomly generated.

*StudyDate:* Fictitious date of the Study.

*Labels:* The labels (Enlarged Cardiomediastinum, Cardiomegaly, Lung Lesion, Lung Opacity, Edema, Consolidation, Pneumonia, Atelectasis, Pneumothorax, Pleural Effusion, Pleural Other, Fracture, Support Devices and No Findings) are indicated in separated columns. The code “1” is assigned for positive mention, “0” for negation, “” for no mention, and “−1” for uncertainty. *No Finding* - Value is 1 if no other label is present, except for support devices.

*ViewPosition:* Radiographic view associated with Patient Position. Defined Terms: AP - Anterior/Posterior; PA - Posterior/Anterior; LL - Left Lateral; RL - Right Lateral; RLD - Right Lateral Decubitus; LLD - Left Lateral Decubitus; RLO - Right Lateral Oblique; LLO - Left Lateral Oblique”. Blank values refer to unavailable View Position information in the DICOM metadata.

*Rows:* Size (number of pixels) in the vertical axis of the image matrix.

*Columns:* Size (number of pixels) in the horizontal axis of the image matrix.

*Manufacturer:* Index for the manufacturer of the CT scanner. The Manufacturer’s name is coded in integers to conceal the actual manufacturer but still allow future research to be conducted on possible biases related to the vendor and/or machine settings.

## Technical Validation

### Automated labeling of the radiology reports

To evaluate effectiveness of the automated labeling procedure, 1000 reports were randomly selected and reviewed by two board-certified radiologists (E.P.R e M.C.B.S) with over 2 years of experience. When necessary, labels were corrected accordingly. The performance of combining NegEx and CheXpert - automated radiology report labelers - is presented in Table 2 with sensitivity (recall), specificity, accuracy, and F1-score compared to ground truth (i.e., agreement between the two radiologists).

**Table 2:** Performance of the automated labeling of the radiology reports.

Findings	Mention			Negation			Uncertainty		
	F1	Recall	Precision	F1	Recall	Precision	F1	Recall	Precision
Atelectasis	0.931	0.900	0.964	0.667	1.000	0.500	0.333	0.500	0.250
Cardiomegaly	0.947	0.986	0.910	0.996	0.993	1.000	0.907	0.975	0.848
Consolidation	0.824	0.824	0.824	0.969	1.000	0.939	N/A	N/A	N/A
Edema	0.800	1.000	0.667	N/A	N/A	N/A	0.889	0.800	1.000
Pleural Effusion	0.925	0.977	0.878	0.992	0.986	0.997	0.308	0.200	0.667
Pneumonia	0.762	0.667	0.889	N/A	N/A	N/A	0.800	0.889	0.727
Pneumothorax	1.000	1.000	1.000	1.000	1.000	1.000	1.000	1.000	1.000
Enlarged Cardiomediastinum	0.857	0.750	1.000	0.990	0.980	1.000	N/A	N/A	N/A
Lung Lesion	0.795	0.861	0.738	0.667	1.000	0.500	0.800	1.000	0.667
Lung Opacity	0.933	0.885	0.986	0.400	1.000	0.250	0.200	0.667	0.118
Pleural Other	0.901	0.865	0.941	N/A	N/A	N/A	0.182	0.100	1.000
Fracture	0.850	0.739	1.000	0.400	0.333	0.500	0.000	0.000	0.000
Support Devices	0.987	0.996	0.978	0.600	0.600	0.600	N/A	N/A	N/A
No Finding	0.821	0.993	0.700	N/A	N/A	N/A	N/A	N/A	N/A

Performance of the automated radiology report labeler (pipeline output from NegEx and BRAX labeler) on a subset of 1,000 reports compared to the labeling agreement between two board-certified radiologists on tasks of mention extraction, negation detection and uncertainty detection, as measured by F1-score, Recall and Precision.

## Usage Notes

Free-text reports are not yet provided in the current version. Future releases shall provide greater volumetry and possibly other metadata for evaluation of social determinants of health. We did not assess potential biases of gender, race or socioeconomic factors in our dataset. Use of the data requires signing a data use agreement that stipulates, among other items, that the user will not share or attempt to re-identify the data. Once approved, data can be directly downloaded from the BRAX Database project on PhysioNet^21,22^ at 10.13026/grwk-yh18.

## Data Availability

The BRAX Labeler code used for the extraction of labels from Brazilian-Portuguese radiology reports is available on Github (https://github.com/edreisMD/BRAX-labeler). To prevent the risk of patient re-identification, the anonymization code is not provided.
